# Gastric duplication cyst in an adult woman: a case report

**DOI:** 10.1093/jscr/rjaf324

**Published:** 2025-06-09

**Authors:** Samuel Hernández-Alvarado, Christian Guadalupe Peña-Gio, Rodrigo Villarreal-Zavala, Misael Gutiérrez-Pérez, Diego Pérez-Valdez, José Rodrigo Alcántara-Dzib, Johny Jefrey Ortiz-Agustín

**Affiliations:** General Surgery Department, Hospital General Regional No. 1 “Ignacio García Téllez” Instituto Mexicano del Seguro Social (IMSS), Calle 34 × 41, No. 439, Colonia Industrial, Z.P. 97150 Mérida, Yucatán, México; General Surgery Department, Hospital General Regional No. 1 “Ignacio García Téllez” Instituto Mexicano del Seguro Social (IMSS), Calle 34 × 41, No. 439, Colonia Industrial, Z.P. 97150 Mérida, Yucatán, México; General Surgery Department, Hospital General Regional No. 1 “Ignacio García Téllez” Instituto Mexicano del Seguro Social (IMSS), Calle 34 × 41, No. 439, Colonia Industrial, Z.P. 97150 Mérida, Yucatán, México; General Surgery Department, Hospital General Regional No. 1 “Ignacio García Téllez” Instituto Mexicano del Seguro Social (IMSS), Calle 34 × 41, No. 439, Colonia Industrial, Z.P. 97150 Mérida, Yucatán, México; General Surgery Department, Hospital General Regional No. 1 “Ignacio García Téllez” Instituto Mexicano del Seguro Social (IMSS), Calle 34 × 41, No. 439, Colonia Industrial, Z.P. 97150 Mérida, Yucatán, México; Pathology Department, Hospital General Regional No. 1 “Ignacio García Téllez” Instituto Mexicano del Seguro Social (IMSS), Calle 34 × 41, No. 439, Colonia Industrial, Z.P. 97150 Mérida, Yucatán, México; Oncology Surgery Department, Hospital General Regional No. 1 “Ignacio García Téllez” Instituto Mexicano del Seguro Social (IMSS), Calle 34 × 41, No. 439, Colonia Industrial, Z.P. 97150 Mérida, Yucatán, México

**Keywords:** gastric duplication, gastric cyst, gastric mass, gastric surgery

## Abstract

Gastric duplication cyst is one of the rarest congenital anomalies of gastrointestinal tract duplications. These are usually diagnosed within the first year of life; however, some cases are diagnosed in adulthood. The diagnosis of these cysts is challenging because symptoms vary depending on their location and are often nonspecific. Definitive diagnosis is achieved through histopathological results. The purpose of this report is to describe a case of gastric duplication in a 71-year-old woman treated at the Regional Hospital in the city of Mérida, Mexico, who underwent exploratory laparotomy for resection of a cyst located in the gastric fundus.

## Introduction

Gastric duplication cyst is a rare congenital anomaly of the gastrointestinal tract and it is especially uncommon in adults [1]. Approximately 67% of the cases are diagnosed during the first year of life, with a reported incidence of 1 in every 4500 births. These cysts may appear anywhere along the gastrointestinal tract from mouth to anus. They most frequently occur in the ileum, followed by jejunum, esophagus, and colon, with gastric duplication being the least common (3.8%) of all gastrointestinal tract duplications [2]. Several theories have been proposed regarding their etiology, including the formation of embryologic enteric diverticula, incomplete separation of notochordal plates, and fusion of longitudinal folds [3]. There are two anatomical forms: cystic (80%) and tubular (20%) and can be single or multiple [4].

Preoperative diagnosis of these congenital anomalies is difficult. The differential diagnoses are broad and include gastrointestinal stromal tumors (GISTs), neuroendocrine tumors, pancreatic heterotopia, pancreatic pseudocysts, and neurogenic tumors. The clinical presentation varies depending on the patient's age and the cyst’s location. Some patients present symptoms due to complications such as intestinal obstruction, infection, bleeding, or perforation [5].

Imaging studies are useful for diagnosis and help define the location of the mass and its relationship with surrounding structures. However, definitive confirmation is made postoperatively through pathology findings [5].

## Case report

**Figure 1:**
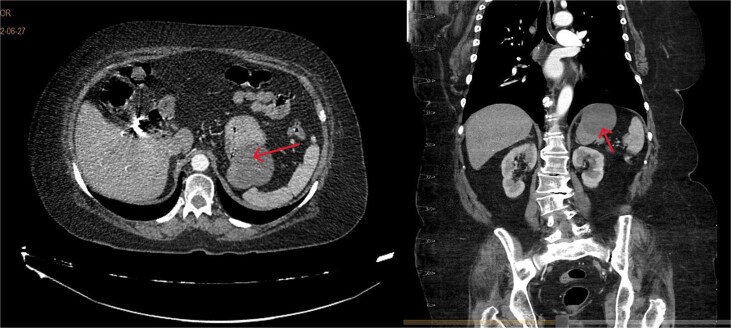
Contrast-enhanced CT, transverse, and coronal sections. Arrows: gastric duplication cyst. A slight displacement of the stomach is observed, without spleen involvement and independent from the pancreas.

A 71-year-old female with a history of systemic arterial hypertension and dyslipidemia under treatment, with previous laparoscopic cholecystectomy and total abdominal hysterectomy. No other relevant medical history. She presented to the emergency department with gastrointestinal symptoms of 2 months' evolution, including postprandial abdominal pain, nausea, and vomiting, resulting in oral intolerance, which required hospital admission. The physical examination was unremarkable. During her stay in the emergency department, various imaging studies were performed, and the computed tomography (CT) scan revealed a hypodense lesion measuring 66 × 50 × 58 mm with thin gastric walls (Fig. 1).

**Figure 2:**
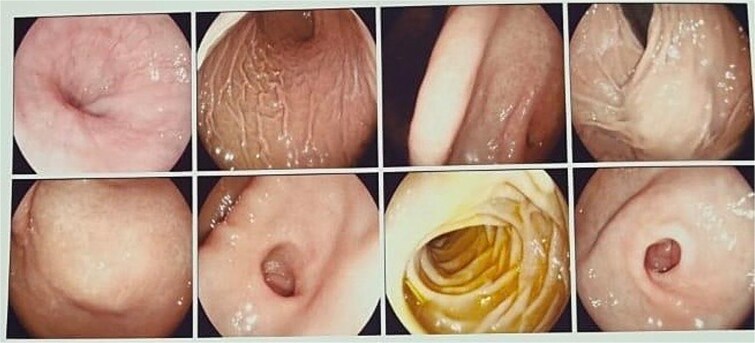
Panendoscopy. Normal gastric mucosa is observed, with extrinsic compression at the level of the fundus and antrum.

**Figure 3:**
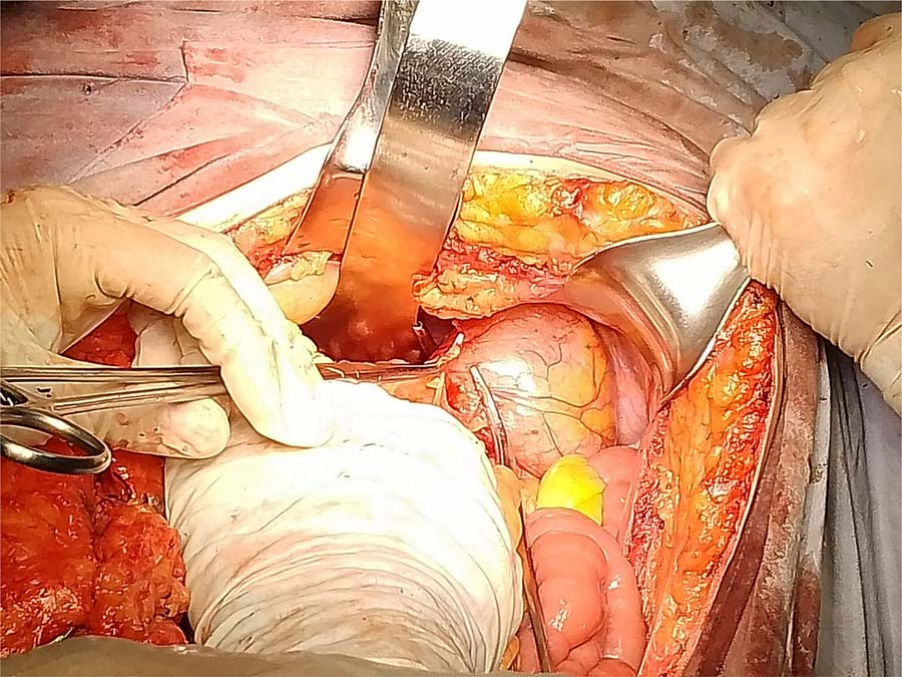
Photograph taken during surgery showing the resected gastric cyst with the surrounding anatomical structures.

**Figure 4:**
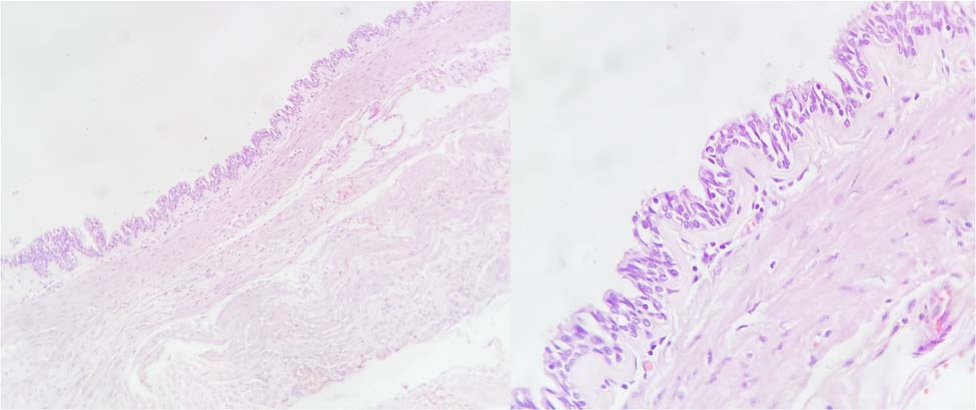
Microscopy of the gastric duplication cyst, hematoxylin–eosin stain. The presence of mucosa and submucosa is observed. Magnified view showing respiratory-type mucosal pattern (pseudostratified columnar epithelium).

Based on the CT findings, a diagnostic protocol was initiated, including blood tests, which showed no abnormalities in tumor markers: CA19.9 was 2.2 U/ml and carcinoembryonic antigen was 2.06 ng/ml. An endoscopic study was also performed, which reported extrinsic compression of the gastric mucosa at the level of the body and antrum (Fig. 2). Given the multiple differential diagnoses and the progression of the patient’s symptoms, a diagnostic and therapeutic exploratory laparotomy was decided and performed on 13 March 2024. Intraoperative findings included multiple omental-wall adhesions and a cystic tumor adhered to the serosa of the gastric fundus measuring approximately 6 × 6 cm (Fig. 3), with accidental rupture of the cystic mass. Complete resection of the lesion was performed, and the specimen was sent for histopathological analysis to establish a definitive diagnosis. The patient had a favorable postoperative course and was discharged after 72 h with outpatient follow-up. During follow-up, no late postoperative complications were observed, and the histopathology report confirmed the diagnosis of gastric duplication cyst (Fig. 4).

## Discussion

Gastric duplications are usually diagnosed during infancy, but in rare cases, they may remain asymptomatic until adulthood, with the diagnosis often made incidentally during imaging studies [6]. Most are non-communicating cysts located on the greater curvature of the stomach, although they may also be found in atypical locations [7].

Preoperative diagnosis in adults is complex due to the lack of specific symptoms and the similarity with other submucosal lesions such as GISTs, neuroendocrine tumors, and pancreatic heterotopia [8]. CT and MRI may reveal a well-defined cystic lesion; however, these are often mistaken for pancreatic cysts or gastric pseudotumors [1]. Endoscopic ultrasound with fine needle aspiration (EUS-FNA) has proven useful for distinguishing between duplication cysts and neoplastic lesions, allowing evaluation of the cyst wall and its contents [9].

Histologically, these cysts show a proper muscular layer and digestive epithelium, mainly gastric mucosa, although they may contain heterotopic epithelium such as respiratory or pancreatic types [4]. In some cases, ectopic gastric mucosa may be functional and secrete acid, leading to complications like ulceration or gastrointestinal bleeding [7].

Although most gastric duplication cysts are benign, there is a low risk of malignant transformation. Cases of adenocarcinoma and neuroendocrine tumors arising in the epithelium of the duplication have been reported [10]. Some studies have documented malignant degeneration in adult patients, highlighting the importance of early surgical resection even in asymptomatic cases [3].

The treatment of choice is complete surgical resection, which can be performed either by open or laparoscopic approach. Segmental resection with gastric reconstruction is preferred when the duplication shares a significant portion of the gastric wall [2]. For small lesions, endoscopic excision has been used successfully, though its use remains controversial due to the risk of perforation and potential malignancy [1].

## Conclusion

Gastric duplications in adults represent a diagnostic challenge due to their atypical presentation and lack of specific symptoms. CT and MRI, along with EUS-FNA, can help differentiate these lesions from other gastric neoplasms. Although malignant transformation is rare, surgical resection remains the best strategy to prevent complications and confirm the histological diagnosis.

This case underscores the importance of recognizing this pathology in adult patients, especially when clinical presentation is atypical. A multidisciplinary evaluation and precise surgical approach enable successful treatment, ensuring timely diagnosis and avoiding complications.
